# Cordycepin induced MA-10 mouse Leydig tumor cell apoptosis by regulating p38 MAPKs and PI3K/AKT signaling pathways

**DOI:** 10.1038/srep13372

**Published:** 2015-08-25

**Authors:** Bo-Syong Pan, Yang-Kao Wang, Meng-Shao Lai, Yi-Fen Mu, Bu-Miin Huang

**Affiliations:** 1 Institute of Basic Medical Sciences, College of Medicine, National Chen Kung University, Tainan, Taiwan, Republic of China; 2 Department of Cell Biology and Anatomy, College of Medicine, National Chen Kung University, Tainan, Taiwan, Republic of China

## Abstract

The p38 MAPKs play important roles in the regulation of balance between cell survival and cell death on the development of various cancers. However, the roles of p38 MAPKs regulating apoptotic effects on Leydig tumor cells remain unclear. In the present study, we showed that cordycepin (3′-deoxyadenosine) selectively induced apoptosis in MA-10 mouse Leydig tumor cells through regulating the p38 MAPK and PI3K/AKT signaling pathways. Cordycepin reduced viability in MA-10, TM4, and NT2/D1 cells, but not cause cell death of primary mouse Leydig cells on moderate concentration. Cordycepin increased reactive oxygen species (ROS) levels, which is associated with the induction of apoptosis as characterized by positive Annexin V binding, activation of caspase-3, and cleavage of PARP. Inhibition of p38 MAPKs activity by SB203580 significantly prevented cordycepin-induced apoptosis in MA-10 cells. Co-treatment with wortmannin or the autophagy inhibitor 3-methyladenine (3-MA) elevated levels of apoptosis in cordycepin-treated MA-10 cells. Moreover, cordycepin activated p53, p21 and TGFß; and downregulated CDK2. The antitumour activity of cordycepin-treated MA-10 cells was significantly distinct in severe combined immunodeficiency (SCID) mice *in vivo*. These results suggested that cordycein is a highly selective treatment to induce MA-10 cells apoptosis via p38 MAPKs signaling.

The Mitogen Activated Protein Kinases (MAPKs) are expressed in all cell types and play key role in gene expression, mitosis, differentiation, proliferation, cell survival, and apoptosis^1^. Up to now, six distinct groups of MAPKs have been identified in mammals: the extracellular signal-regulated kinases ERK 1/2, ERK 3/4, ERK5, ERK 7/8, the Jun N-terminal kinases JNK1/2/3 and the p38 MAPKs p38α/β/γ/δ^2^. The p38 MAPKs play an important role in the regulation of balance between cell survival and cell death on the development of various cancers^3^. Several reports presented the pro-apoptotic role of p38 MAPKs; for example, p38 MAPKs activate apoptosis through oxidative stress by generating reactive oxygen species (ROS)^4^. Moreover, p38α induces apoptosis through up-regulation of the pro-apoptotic proteins Fas and Bax and down regulation of the activity of the ERK and Akt survival pathways^5^.

Recent studies have provided new insight on the control of the balance of autophagy and apoptosis through p38 MAPKs signaling in response to chemotherapeutic agents^6^. The p38 MAPKs are major determinant of the balance between p53-dependent apoptosis and autophagy triggered by 5-fluorouracil^7^. The p53 induces expression of genes involved in both death receptor dependent (CD95 and DR5) and mitochondrion-dependent (PUMA, Bax and Bid) apoptotic pathways^8^. Among p53 binding partners, cyclin-dependent kinase inhibitor 1A (CDKN1A, also known as p21) is an important part of the p53 response. p21 could inhibit cyclin-dependent kinase 1 and 2 (CDK1 and 2) and thereby block mitosis in response to DNA damage^9^. In the cytoplasm, p21 blocks apoptosis by inhibiting caspase-3^10^.

Testicular cancer is one of the most common cancers in men between 15 and 35 years old with about 8,000 cases found in the United States annually^11^. Most (95%) testicular tumors are of germ cell origin, and about 5% are of Sertoli cell or Leydig cell (non–germ cell) origin. Leydig cell tumors are the most prevalent hormone-producing tumors of the testis^12^. They account for 1 to 3 percent of all testicular tumors and show two peaks in age incidence: between 5–10 years and 30–35 years^13^. They are usually benign, but 10 percent of tumors in adults are malignant and can metastasize^14^.

Approximately 80% of patients with metastatic disease can now expect long term disease free survival^15^. Though usually benign with good response to treatment, patients’ quality of life is inevitably impaired. Since the expected life span is around 50 years after successful treatment, the focus of therapeutic strategy is to avoid long term toxicity^16^. Fertility is an important issue that has been addressed by several studies^17^,^18^,^19^. A recent study has shown that the loss of the tumor bearing testicle leads to further deterioration of spermatogenetic function^20^. Moreover, the use of cisplatin-based chemotherapy is associated with a dose dependent and, in part, irreversible impairment of fertility^21^. Thus, it is important to develop new chemotherapeutic agents that effectively inhibit testicular cancer without having off-target effects.

Cordycepin (3'-deoxyadenosine) is a major bioactive component found in *Cordyceps sinensis*, which has a wide range of biological effects in the regulation of steroidogenesis, inflammation, and platelet aggregation^22^,^23^,^24^,^25^,^26^. Moreover, cordycepin has been shown to exert a large variety of anti-tumor abilities^27^,^28^,^29^,^30^. However, few studies have shown that cordyepin induces apoptotic effect thought the regulation of the p38 MAPKs and PI3K/AKT signaling pathways.

In the current work, we showed that cordycepin reduced viability in MA-10, TM4, and NT2/D1 cells. Interestingly, moderate concentration of cordycepin did not cause cell death in primary mouse Leydig cells. Inhibition of p38 MAPKs activity in MA-10 cells is abrogated the anticancer effect of cordycepin. These results indicate an important role for the cordycein-induced MA-10 cells apoptosis via mediating p38 MAPKs signaling.

## Results

### Cordycepin induced apoptosis in MA-10 mouse Leydig tumor cells

**Figure 1:**
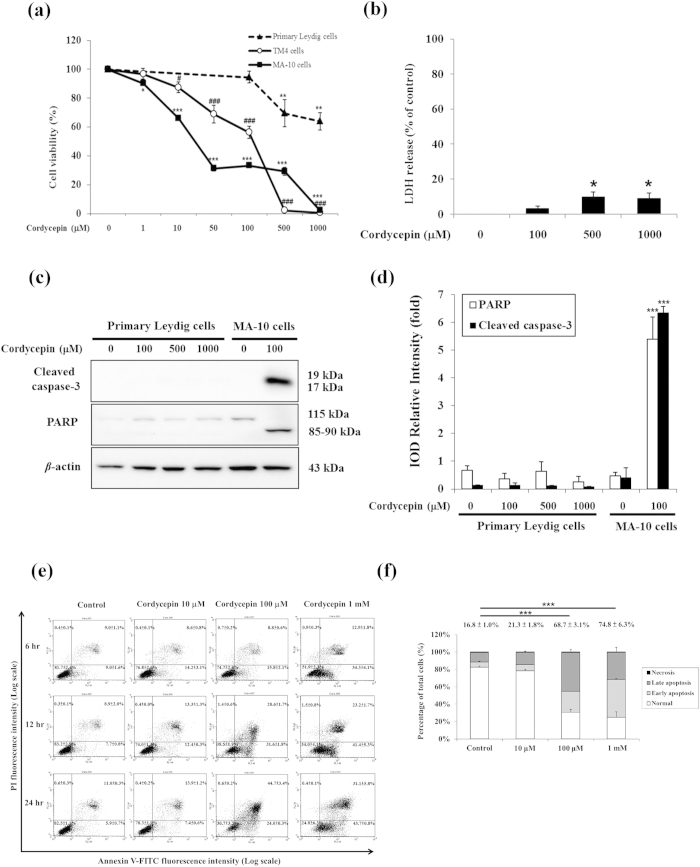
Cordycepin induced apoptosis of testicular tumor cells. (**a**) Primary mouse Leydig cells, MA-10 mouse Leydig tumor cells, and TM4 Sertoli tumor cells were cultured in medium with or without cordycepin for 24 h. The MTT assay was performed to assess cell viability. (**b**) Primary mouse Leydig cells were cultured in medium with or without cordycepin for 24 h. The LDH release assay was performed to assess cell death. (**c**) Primary mouse Leydig cells and MA-10 cells were treated with or without cordycepin for 12 h. Levels of cleaved caspase-3 (17/19 kDa) and cleaved PARP (85–90 kDa) were detected by western blotting. (**d**) Integrated optical densities (IODs) of protein bands in (**c**) associated with cleaved caspase-3 and cleaved PARP are shown. Data normalized to β-actin. (**e**) MA-10 cells were treated with or without cordycepin for indicated times and stained with annexin V and propidium iodide (PI). Flow cytometry was used to determine the fractions of viable cells (annexin V^−^, PI^−^; lower left quadrant), necrotic cells (annexin V^−^, PI^+^; upper left), early apoptotic cells (annexin V^+^, PI^−^; lower right), and late apoptotic cells (annexin V^+^, PI^+^; upper right). (**f**) Plot of cell viability from flow cytometric analysis at 24 h. Data represent mean ± SEM for four independent experiments. *or ^**#**^*p* < 0.05; **or ^**##**^*p* < 0.01; ***or ^**###**^*p* < 0.001.

The MTT test was used to investigate the effect of cordycepin on cell viability in primary mouse Leydig cells, MA-10 mouse Leydig tumor cells, and TM4 Sertoli tumor cells (Fig. 1a). After 24 h of cordycepin treatment (10 μM to 1 mM), cell death was seen in MA-10 and TM4 tumor cells in a dose-dependent manner, but was only seen in primary mouse Leydig cells at the highest doses of cordycepin (500 and 1,000 μM). To further test the effect of cordycepin on cell death in mouse Leydig cells, we used the LDH release assay and western blotting. Treating primary mouse Leydig cells with 100 μM cordycepin did not release a significant amount of LDH compared to control (Fig. 1b). Western blot analysis showed that cordycepin upregulated cleavage of caspase-3 and PARP in MA-10 cells (*p* < 0.001), but did not significantly affect levels of cleaved proteins in primary mouse Leydig cells (*p* > 0.05; Fig. 1c,d). These results indicate that 100 μM cordycepin did not cause cell death in primary mouse Leydig cells. However, it should be noted that high concentration of cordycepin (1,000 μM) did induce cell death in primary mouse Leydig cells.

To determine the mode of cell death induced by cordycepin in MA-10 cells, we labeled cells with annexin V and PI and used flow cytometry to distinguish early apoptotic (annexin V^+^, PI^−^) and late apoptotic (annexin V^+^, PI^+^) cells from viable cells (annexin V^−^, PI^−^) and necrotic cells (annexin V^−^, PI^+^). Cells were treated with cordycepin (10 μM, 100 μM, or 1 mM) for 6, 12, or 24 h (Fig. 1e). At 24 h, the percentage of apoptotic cells (both early and late) for control, 100 μM cordycepin, and 1 mM cordycepin was 16.8 ± 1.0%, 68.7 ± 3.1%, and 74.8 ± 6.3%, respectively (*p* < 0.001; Fig. 1f). These results clearly illustrate that cordycepin induced apoptosis in MA-10 mouse Leydig tumor cells.

Pluripotent embryonal carcinomas (EC) are the malignant counterparts to embryonic stem cells and are considered the stem cells of testicular germ cell tumors (TGCT)^31^. The data indicated that cordycepin significantly reduced cell viability in NT2/D1 cells as etoposide effects (Figure S1A). NT2/D1 cells were treated with cordycepin for 24 h, and upregulated protein levels of cleavage of caspase-3 and PARP were assessed by western blotting (Figure S1B). The G2M and subG1/G0 phase cells number were markedly increased among cordycepin-treated NT2//D1 cells, as shown in Figure S1C and S1D. These observations suggest that cordycepin significantly suppressed the viability of testicular cancer cells, including testicular germ cell tumors, MA-10 mouse Leydig tumor cells, and TM4 Sertoli tumor cells.

### Cordycepin induced tumor cell apoptosis through caspase signaling

**Figure 2:**
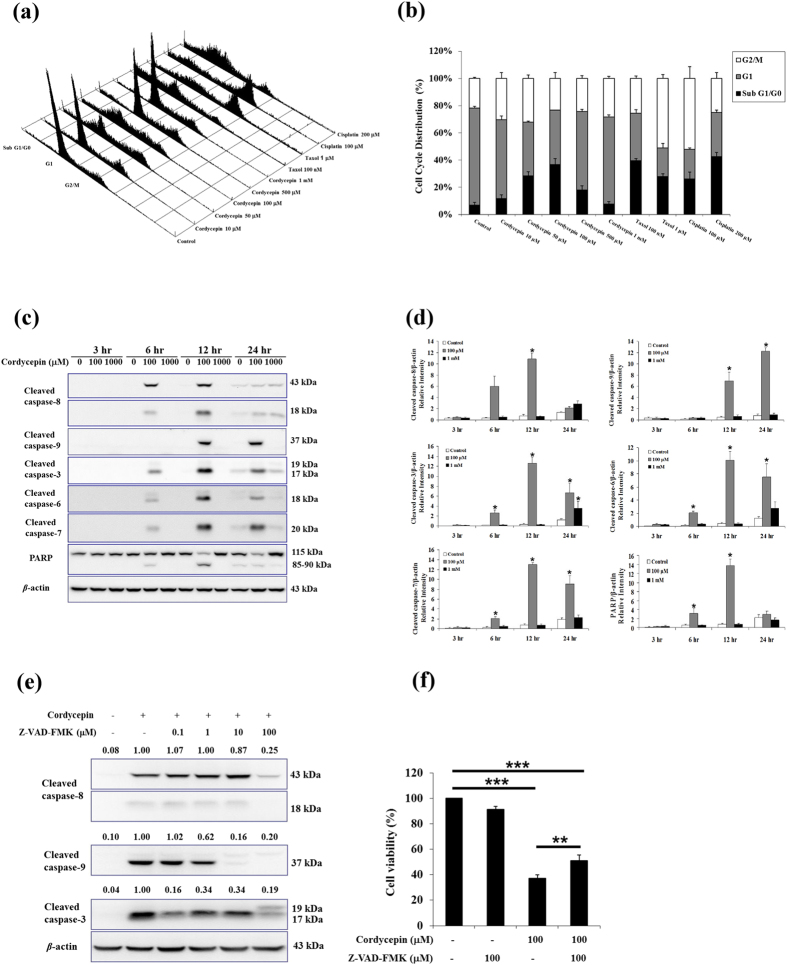
Cordycepin induced apoptosis of MA-10 cells through caspase signaling. (**a**) MA-10 cells were treated with or without cordycepin, taxol, and cisplatin at the indicated dosages at 24 h, and stained with propidium iodide (PI). Flow cytometry was used to determine the fractions of sub G1/G0, G1, and G2/M phases. (**b**) Plot of cell cycle distribution from flow cytometric analysis from (**a**). (**c**) MA-10 cells were treated with or without cordycepin for the indicated times. Cleaved caspase-3 (17 and 19 kDa), –6 (18 kDa), –7 (20 kDa), –8 (43 and 18 kDa), and –9 (37 kDa), and cleaved PARP (85–90 kDa) were detected by western blotting. (**d**) Integrated optical densities of protein bands in (**c**). Data were normalized to β-actin. (**e**) MA-10 cells were pretreated with the general caspase inhibitor Z-VAD-FMK for 1 h, then 100 μM cordycepin was added and cells were treated for 12 h. Cleaved caspases were detected by western blotting. Band intensities relative to β-actin are shown above each blot. (**f**) Cell viability was analyzed using the MTT assay after cells were treated with the general caspase inhibitor Z-VAD-FMK and 100 μM cordycepin for 24 h. Data represent mean ± SEM for three independent experiments. *p < 0.05, **p < 0.01, ***p < 0.001.

We next examined the effect of different dosages of cordycepin on cell cycle distribution in MA-10 cells with flow cytometry assay (Fig. 2a), and found that cordycepin at 10 and 50 μM started to increase subG1/G0 phase cells number with maximal effect by 100 μM, but did not significantly affect by 1 mM (Fig. 2b). Taxol and cisplatin, the clinically used anti-cancer drugs^32^, were used as a positive control. Cordycepin, taxol and cisplatin had similar effects on subG1/G0 phase in MA-10 cells (Fig. 2b). To characterize the mechanisms by which cordycepin induced apoptosis, we analyzed the expression of proteins that activated caspase pathways. MA-10 cells were treated with cordycepin (control, 100 μM, or 1 mM) for 3, 6, 12, or 24 h, and levels of cleaved caspase-3, –6, –7, –8, –9, and PARP were assessed by western blotting (Fig. 2c). Cordycepin (100 μM) induced cleavage of caspase-3, –6, –7, –8, and PARP after 6 h treatment, with maximal cleavage at 12 h (Fig. 2d). Cordycepin (100 μM) induced cleavage of caspase-9 as well, but only after 12 h treatment, with maximal cleavage at 24 h (*p* < 0.05). Interestingly, the only effect seen with 1 mM cordycepin was on caspase-3 cleavage after 24 h (*p* < 0.05). These data imply that 100 μM and 1 mM cordycepin may have activated different signaling pathways to induce apoptosis of MA-10 cells.

To confirm the cordycepin-induced caspase signaling cascades in MA-10 cells, the following caspase inhibitors were tested: Z-VAD-FMK (general caspase inhibitor), Z-IETD-FMK (caspase-8 inhibitor), and Z-LEHD-FMK (caspase-9 inhibitor). MA-10 cells were treated with caspase inhibitors for 1 h and then cordycepin (100 μM) was added and cells were treated for an additional 12 h. The general caspase inhibitor Z-VAD-FMK significantly attenuated cleavage of caspase-3, –8, and –9 (*p* < 0.05; Fig. 2e), and specific inhibitors also affected levels of the appropriate cleaved caspases (*p* < 0.05; Figure S2A,B). Moreover, the MTT assay was used to determine whether the general caspase inhibitor could rescue the viability of MA-10 cells treated with cordycepin. Cordycepin (100 μM) reduced MA-10 cell viability to 37% (relative to control) after 24 h of treatment (*p* < 0.001), but only Z-VAD-FMK improved the viability of cordycepin-treated MA-10 cells by 14% (*p* < 0.01; Fig. 2f and Figure S2C). Results also show that caspase-3 siRNA significantly reduced cleaved caspase-3 expression and recovered cell viability with cordycepin treatment in MA-10 cells (*p* < 0.05; Figure S2D,F). These results suggest that activation of caspase pathways was important for cordycepin to induce apoptosis in MA-10 mouse Leydig tumor cells.

### Cordycepin induced apoptosis by activating p38 signaling

The caspase signaling pathways involved in MAPKs activate apoptosis^1^. We therefore examined the effect of cordycepin on phosphorylation of ERK, JNK, and p38 by western blotting. A high dose of cordycepin (1 mM) promoted phosphorylation of ERK with the optimal effects seen after 5 min, 15 min, 6 h, 12 h or 24 h, while intermediate time points (30 min, 1 h, or 3 h) exhibited minor effect (*p* < 0.05; Figure S3A,B). Although there is no statistical difference (Figure S3B), an increasing trend of phosphorylated ERK blotting could be observed by 100 μM cordecypin at 6 hr treatment (Figure S3A). Moreover, treatment with 100 μM cordycepin for 12 h or 1 mM cordycepin for 6, 12, or 24 h induced phosphorylation of JNK (*p* < 0.05), and treatment with 100 μM cordycepin for 12 h or 1 mM cordycepin for 12 or 24 h induced phosphorylation of p38 (*p* < 0.05; Figure S3A,B).

**Figure 3:**
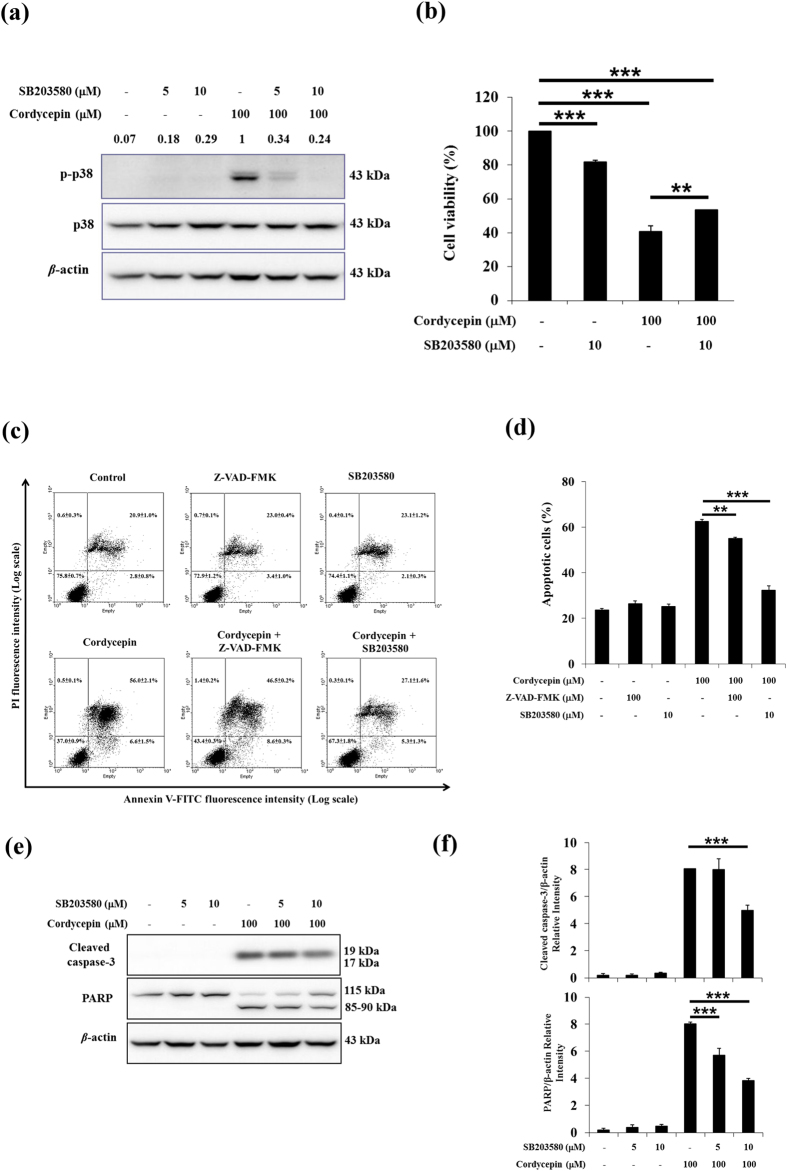
Cordycepin induced apoptosis of MA-10 cells by activating p38 signaling pathways. (**a**) MA-10 cells were pretreated with p38 inhibitor SB203580 for 1 h, then 100 μM cordycepin was added and cells were treated for 12 h. Total and phosphorylated proteins were detected by western blotting. Band intensities relative to β-actin are shown above each blot. (**b**) Cell viability was analyzed using the MTT assay after cells were treated with p38 inhibitor SB203580 and 100 μM cordycepin for 24 h. (**c**) MA-10 cells were pretreated with general caspase inhibitor Z-VAD-FMK and p38 inhibitor SB203580 for 1 h, then 100 μM cordycepin was added and cells were treated for 24 h and stained with annexin V and propidium iodide (PI). Flow cytometry was used to determine the fractions of viable cells (annexin V−, PI−; lower left quadrant), necrotic cells (annexin V−, PI+; upper left), early apoptotic cells (annexin V+, PI−; lower right), and late apoptotic cells (annexin V+, PI+; upper right). (**d**) Plot of apoptotic cells from flow cytometric analysis at 24 h. (**e**) MA-10 cells were pretreated with p38 inhibitor SB203580 for 1 h, then 100 μM cordycepin was added and cells were treated for 12 h. Levels of cleaved caspase-3 (17/19 kDa) and cleaved PARP (85–90 kDa) were detected by western blotting. Band intensities relative to β-actin are shown above each blot. (**f**) Integrated optical densities of protein bands in (**e**). Data were normalized to β-actin. Data represent mean ± SEM from three independent experiments. *p < 0.05, **p < 0.01, ***p < 0.001.

To confirm whether MAPK signaling pathways were directly activated by cordycepin, MA-10 cells were pre-treated with inhibitors of ERK (PD98059), JNK (SP600125), and p38 (SB203580) 1 h, then 100 μM cordycepin was added and cells were treated for an additional 12 h. Addition of inhibitors significantly reduced cordycepin-induced elevation of p-ERK, p-JNK, and p-p38 (*p* < 0.05; Fig. 3a and Figure S3C,D). To elucidate which MAPK signaling mediated cordycepin-induced apoptosis, the MTT assay was performed to determine cell viability. Treating MA-10 cells with 100 μM cordycepin alone for 24 h reduced cell viability to 40% of control. Inhibition of p38 partially restored the viability of cordycepin-induced cell death in MA-10 cells (*p* < 0.01; Fig. 3b), but not JNK or ERK inhibitors (Figure S3E).

To determine whether activation of p38 correlates to cordycepin-induced MA-10 cell apoptosis, we labeled cells with annexin V and PI and used flow cytometry. MA-10 cells were pre-treated with inhibitors of general caspase (Z-VAD-FMK) and p38 (SB203580) 1 h, then 100 μM cordycepin was added and cells were treated for an additional 24 h. Treating MA-10 cells with Z-VAD-FMK and SB203580 did not increase a significant amount of apoptotic cells and change morphology compared to control (*p* > 0.05; Fig. 3c,d and Figure S3F). The general caspase inhibitor significantly reduced amount of apoptotic cells associated with 100 μM cordycepin treatment, but only pretreatment with p38 inhibitor restored control levels of apoptotic cells (*p* < 0.001; Fig. 3c,d). Western blotting for cleaved caspase-3 and PARP demonstrates that the target of cordycepin-induced caspase activation is attenuated when the p38 inhibitor SB203580 is added 1 h prior to cordycepin treatment (*p* < 0.001; Fig. 3e,f). These data indicate that cordycepin exerted its effect in cordycepin-induced MA-10 cell apoptosis through p38 MAPKs signaling.

### Cordycepin induced apoptosis and autophagy by regulating PI3K/AKT/mTOR signaling

**Figure 4:**
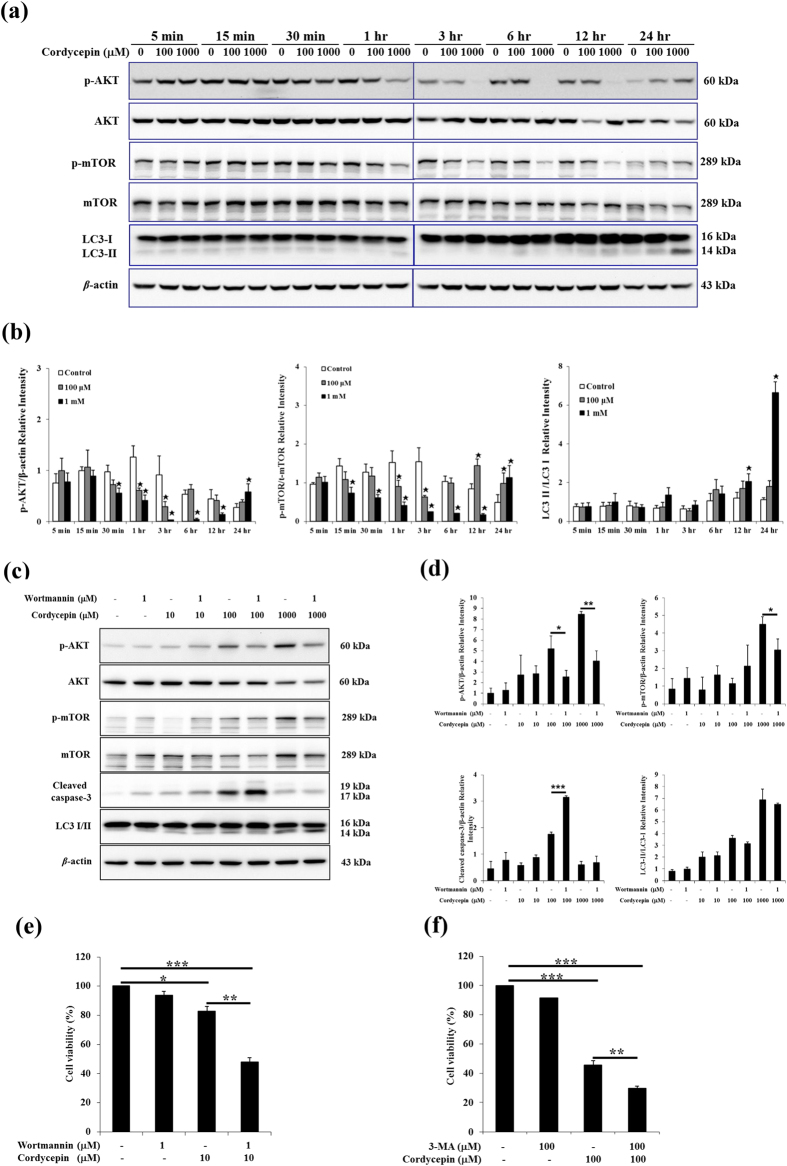
Cordycepin induced apoptosis and autophagy in MA-10 cells by regulating PI3K/AKT/mTOR signaling pathways. (**a**) MA-10 cells were treated with or without cordycepin for the indicated times. Total and phosphorylated AKT (60 kDa) and mTOR (289 kDa), as well as total LC3-I/II (16 and 14 kDa), were detected by western blotting. (**b**) Integrated optical densities of protein bands in (**a**). Data were normalized to β-actin. (**c**) MA-10 cells were pretreated with the PI3K/AKT inhibitor wortmannin for 30 min, then cordycepin was added and cells were treated for 24 h. Total and phosphorylated m-TOR (289 kDa) and AKT (60 kDa), as well as cleaved caspase-3 (17 and 19 KDa), and LC3 I/II (14 and 16 kDa) were detected by western blotting. (**d**) Integrated optical densities of proteins bands in (**c**). Data were normalized to β-actin. (**e**) Cell viability was analyzed using the MTT assay after cells were treated with wortmannin and cordycepin for 24 h. (**f**) Cell viability was analyzed using the MTT assay after cells were treated with 3-methyladenine (3-MA) and cordycepin for 24 h. Data represent mean ± SEM from three independent experiments. *p < 0.05, **p < 0.01, ***p < 0.001.

PI3K/AKT/mTOR signaling pathways and related signaling networks could promote cell growth and survival^33^. To investigate whether cordycepin could regulate PI3K/AKT/mTOR pathways to induce apoptosis in MA-10 tumor Leydig cells, we first examined levels of AKT and mTOR by western blotting. Treatment of MA-10 cells with 100 μM cordycepin for 1 or 3 h or 1 mM cordycepin for 30 min to 12 h significantly reduced phosphorylation of AKT (*p* < 0.05; Fig. 4a,b). Similar regimens of cordycepin treatment also reduced phosphorylation of mTOR in MA-10 cells (*p* < 0.05; Fig. 4a,b). However, levels of p-AKT were elevated (relative to controls) after 24 h of 1 mM cordycepin treatment, whereas p-mTOR levels were elevated after 12 or 24 h of 100 μM cordycepin treatment or 24 h of 1 mM cordycepin treatment. These results demonstrated a bimodal response to cordycepin treatment in PI3K/Akt/mTOR signaling.

To determine whether PI3K signaling pathways contribute to cordycepin-induced apoptosis of MA-10 cells, cells were pretreated with the PI3K/AKT inhibitor wortmannin (1 μM) for 1 h prior to cordycepin (10 μM to 1 mM) treatment, and cells were treated for an additional 24 h. The PI3K/AKT inhibitor significantly reduced levels of p-AKT associated with 100 μM or 1 mM cordycepin treatment, as well as levels of p-mTOR associated with 1 mM cordycepin treatment (*p* < 0.05; Fig. 4c,d). Interestingly, treatment with wortmannin and cordycepin for 24 h increased expression of cleaved caspase-3 (*p* < 0.05; Fig. 4c,d). The MTT assay was therefore used to determine whether PI3K/AKT signaling affected the viability of MA-10 cells treated with cordycepin. The PI3K/AKT inhibitor further reduced the viability of cells in the presence of 10 μM cordycepin for 24 h (*p* < 0.01; Fig. 4e). Moreover, we have published that blocking phosphorylation of AKT by wortmannin further decreased cordycepin-induced cell viability^24^, suggesting the involvement of PI3K/Akt pathway in the regulation of cordycepin-induced apoptosis in MA-10 cells.

After MA-10 cells were treated with 1 mM cordycepin for 12 or 24 h, LC3 II upregulation was detected (*p* < 0.05; Fig. 4a,b), suggesting that autophagy was involved in this apoptotic response. The MTT assay was therefore used to determine whether autophagy affected the viability of MA-10 cells treated with cordycepin. The autophagy inhibitor 3-methyladenine further reduced the viability of cells in the presence of 100 μM cordycepin for 24 h (*p* < 0.01; Fig. 4F). Thus, the inactivation of PI3K/AKT/mTOR signaling pathways and upregulation of LC3 II, which is related to autophagy, may be involved in cordycepin-induced apoptosis of MA-10 cells.

### Cordycepin led to ROS accumulation

**Figure 5:**
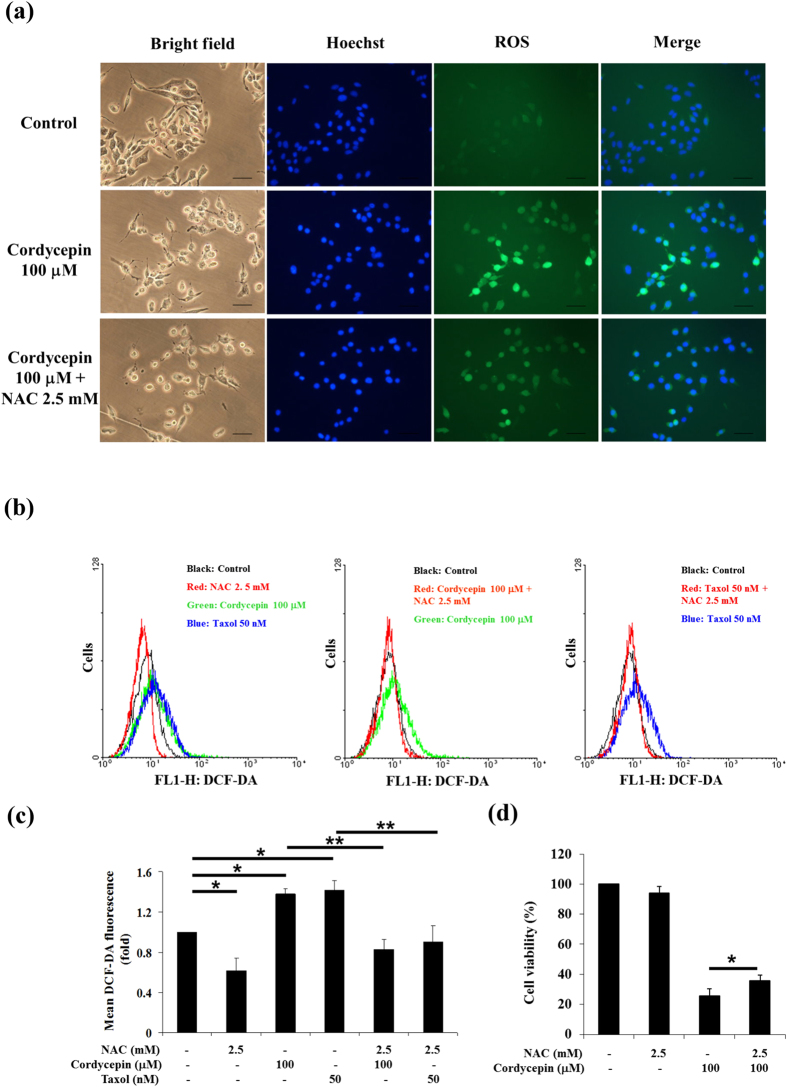
Cordycepin stimulated ROS accumulation in MA-10 cells. (**a**) MA-10 cells were treated with vehicle only (control), with cordycepin (100 μM) alone, or with cordycepin (100 μM) and *N*-acetyl-l-cysteine (NAC; 2.5 mM). Cells were pretreated with NAC for 1 h, followed by cordycepin for 3 h. DNA was labeled using Hoechst stain (blue) and ROS were labeled using DCF-DA (green). (**b**) MA-10 cells were pretreated with 2.5 mM NAC for 1 h, followed by 100 μM cordycepin or 50 nM taxol for 3 h. DCF-DA fluorescence was detected by flow cytometry. (**c**) Quantitation of DCF-DA fluorescence. (**d**) Cell viability was analyzed using MTT assay after cells were treated with NAC followed by 100 μM cordycepin for 24 h. Data were normalized to control and represent mean ± SEM for three independent experiments. *p < 0.05, **p < 0.01.

We next asked whether cordycepin affected the generation of ROS in MA-10 mouse Leydig tumor cells. MA-10 cells were preincubated with 2.5 mM *N*-acetyl-l-cysteine (NAC) for 1 h, then 100 μM cordycepin was added and cells were treated for an additional 3 h. ROS production was determined by fluorescence microscopy (Fig. 5a) and flow cytometry using DCF-DA (Fig. 5b). Treatment with cordycepin alone for 3 h increased ROS levels, but pretreatment with NAC fully reversed this effect (Fig. 5a,b). Quantitative analyses further showed that 100 μM cordycepin alone increased ROS levels by 1.4-fold after 24 h treatment (*p* < 0.05), but pretreatment with NAC (2.5 mM) restored control levels of ROS (*p* < 0.01; Fig. 5c). Taxol, which is known to induce ROS^34^, was used as a positive control. Taxol and cordycepin had similar effects on ROS in MA-10 cells (*p* < 0.01; Fig. 5b,c). To elucidate which ROS accumulation mediated cordycepin-induced apoptosis, the MTT assay was performed to determine cell viability. Results show that NAC significantly recovered cell viability with cordycepin treatment in MA-10 cells (*p* < 0.05; Fig. 5d). These data strongly suggest that an important step involved in cordycepin-induced apoptosis of MA-10 mouse Leydig tumor cells is generation of ROS.

### Cordycepin induced apoptosis by regulating CDK activity through the p53 and TGFβ signaling pathways

**Figure 6:**
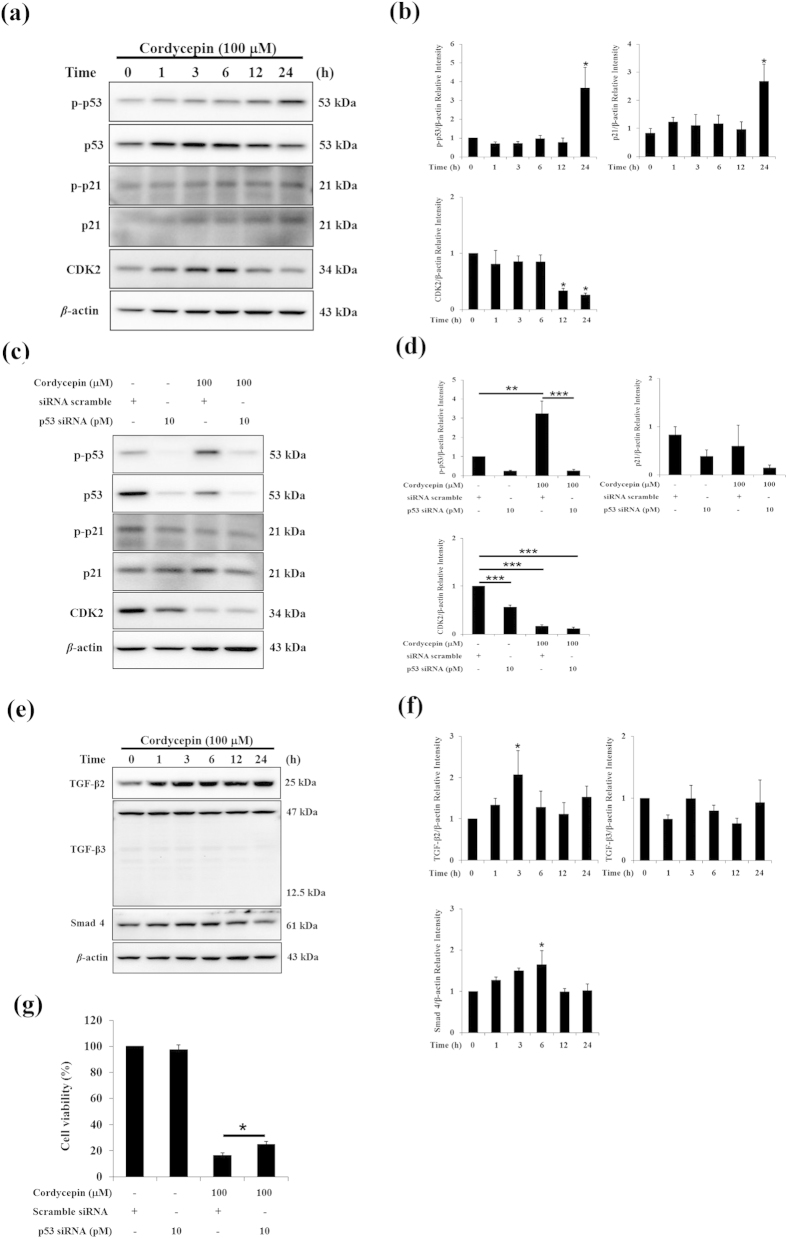
Cordycepin induced apoptosis of MA-10 cells by regulating CDKs through the p53 and TGFβ pathways. (**a**) MA-10 cells were treated with or without cordycepin for the indicated times. Total and phosphorylated p53 (53 kDa) and p21 (21 kDa), as well as total CDK2 (34 kDa), were detected by western blotting. (**b**) Integrated optical densities of protein bands in (**a**). (**c**) MA-10 cells were treated with 100 μM cordycepin and/or p53 siRNA (10 pM) for 24 h. Total and phosphorylated p53 and p21, as well as total CDK2, were detected by western blotting. (**d**) Integrated optical densities of protein bands in (**c**). (**e**) MA-10 cells were treated with or without cordycepin for the indicated times. TGFβ2 (25 kDa), TGFβ3 (12.5 and 47 kDa), and Smad4 (61 kDa) were dete**c**ted by western blotting. (**f**) Integrated optical densities of protein bands in (**e**). (**g**) Cell viability was analyzed using the MTT assay after cells were treated with 100 μM cordycepin and/or p53 siRNA (10 pM) for 24 h. Data represent mean ± SEM for three independent experiments each. *p < 0.05, **p < 0.01, ***p < 0.001.

The p53 signaling pathway plays important roles in regulating cell growth and apoptosis^35^. We speculated whether cordycepin could regulate p53 and downstream components of the p53 pathway to induce apoptosis in MA-10 cells. After 24 h treatment, cordycepin (100 μM) upregulated levels of p-p53 and p21 (*p* < 0.05), but downregulated CDK2 (*p* < 0.05; Fig. 6a,b). To further test the role of p53 in cordycepin-induced apoptosis of MA-10 cells, we used a siRNA approach to downregulate p53 levels. Knockdown of p53 reduced levels of p-p53 in cordycepin-treated MA-10 cells (*p* < 0.001; Fig. 6c,d), as expected. However, it did not affect p21 or CDK2 levels (*p* > 0.05; Fig. 6c,d).

Since TGFβ signals could regulate cell apoptosis in the context of the cell cycle^36^, TGFβ signaling pathways may regulate CDK2 activity and contribute to MA-10 cell apoptosis. After cells were treated with cordycepin for 3 or 6 h, levels of TGFβ2 and Smad4 were upregulated, but TGFβ3 was not affected (*p* > 0.05; Fig. 6e,f). Moreover, the MTT assay was used to determine whether the blocked p53 signaling could rescue the viability of MA-10 cells treated with cordycepin. Results show that p53 siRNA recovered cell viability with cordycepin treatment in MA-10 cells (*p* < 0.05; Fig. 6g). These results suggest that the p53 and TGFβ signaling pathways may play critical roles in cordycepin-induced cell cycle arrest and apoptosis of MA-10 mouse Leydig tumor cells.

### Cordycepin inhibited tumor growth in an allograft model of testicular cancer

**Figure 7:**
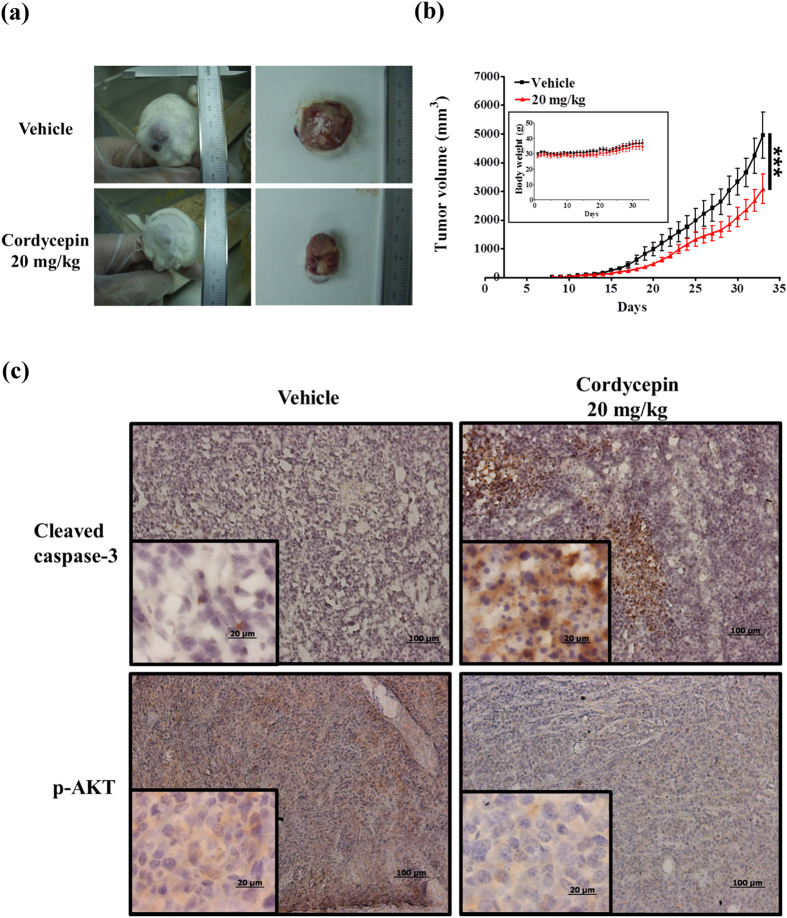
Cordycepin inhibited tumor growth in an allograft model of testicular cancer. SCID mice were injected with MA-10 cells and then treated with PBS (6 mice) or 20 mg/kg cordycepin (6 mice) once daily for 26 d. (**a**) Tumors on the back of SCID mice treated with vehicle or cordycepin. Dissected tumors are shown on the right. (**b**) Tumor volumes plotted against time for control and cordycepin-treated mice. Body weights are also shown (inset). (**c**) Tumors were collected after 33 d, and expression of cleaved caspase-3 and p-AKT was evaluated by immunohistochemistry. ***p < 0.001.

Based on the ability of cordycepin to induce apoptosis of MA-10 mouse Leydig tumor cells *in vitro*, we next tested the *in vivo* effect of cordycepin using tumor allografts in SCID mice. Male SCID mice were injected subcutaneously with MA-10 cells. After 1 week, mice were given daily injections of vehicle or cordycepin (0.1% in PBS; 20 mg/kg). Antitumor effects were observed in tumor-bearing mice treated with cordycepin compared with controls (*n* = 6 each group; Fig. 7a). Animals treated with cordycepin exhibited a significant decrease in tumor volume compared with control mice beginning on day 21 (616.5 vs. 1201 mm^3^; *p* < 0.05; Fig. 7b), with maximal differences seen on day 33 (3091 vs. 4956 mm^3^; *p* < 0.001; Fig. 7b). Importantly, there was not a significant difference in body weight between control and cordycepin-treated mice, indicating that cordycepin treatment had an acceptable safety profile (*p* > 0.05; Fig. 7b inset). Moreover, immunohistochemical analysis of tumors revealed increased levels of cleaved caspase-3 and decreased levels of p-AKT in cordycepin-treated tumors compared to control mice, indicating cellular apoptosis (Fig. 7c). Thus, cordycepin effectively inhibited tumor growth in both *in vitro* cellular system and *in vivo* animal model of tumorigenesis.

## Discussion

As Leydig tumors occur most frequently on children and patients will expect long term survival, elucidation of new chemotherapeutic agents without having off-target effects may benefit patients suffering from Lydig tumors and other testicular cancers. In this study, our results indicated that cordycepin reduced the cell viability in MA-10, TM4, and NT2/D1 cells; activated caspases, induced cell cycle arrest, regulated p38 MAPKs signaling, increased ROS levels, and inactivated PI3K/AKT signaling in MA-10 mouse Leydig tumor cells. These results indicate an important role for the cordycein to induce MA-10 cell apoptosis via mediating p38 MAPKs signaling.

We have found that 100 μM or 1 mM cordycepin induced cleavage of caspase proteins in MA-10 cells. However, different doses of cordycepin affected different caspases. Previous studies have shown that a single factor can activate different patterns of caspase proteins under different treatment regimens (i.e., temporal or dosage variations)^37^, as we have seen here. We further showed that caspase inhibitors improved the viability of cordycepin-treated MA-10 cells, confirming that cordycepin activated caspase cascades to induce the apoptosis of MA-10 cells.

Inhibiting of p38 MAPKs effectively downregulated apoptosis of MA-10 cells treated with cordycepin. Thus, the p38 pathway may play a critical role in cordycepin-induced apoptosis of MA-10 mouse Leydig tumor cells. Previous studies have shown that p38 is important for induction of apoptosis in human breast cancer cells and colon cancer cells^38^,^39^, which supports our current observation.

PI3K/mTOR inhibition increases the effectiveness of therapeutic drugs in several cancers^40^. Here, cordycepin suppressed levels of p-AKT and p-mTOR. Indeed, cordycepin combined with a PI3K/AKT inhibitor promoted cleavage of caspase-3 in MA-10 cells, indicating that the PI3K/AKT/mTOR signaling pathways also play an important role in cordycepin-induced apoptosis of MA-10 cells. Interestingly, levels of p-AKT and p-mTOR were significantly higher when cells were treated with 1 mM cordycepin for 24 h. This suggests that a protective effect may be activated by high dosages of cordycepin. This warrants further investigation, as these studies may reveal mechanisms by which cells develop resistance to chemotherapeutic agents.

Autophagy is essential for cancer cells to survive under conditions of nutrient starvation, hypoxia, or chemotherapeutic stress^41^. Here, high doses of cordycepin upregulated levels of LC3 II, which indicates autophagy. In addition, the inhibition of autophagy increased the cytotoxic effects of cordycepin in MA-10 cells. These results again suggest that a protective effect (this time related to autophagy) may have been activated in cells exposed to high levels of cordycepin.

Excessive generation of ROS, however, causes mitochondrial dysfunction related to apoptosis^42^. In the current study, excessive generation of ROS was detected in cordycepin-treated MA-10 cells, but treatment with NAC inhibited ROS production and generated cells with normal morphology. Thus, our data show that cordycepin-dependent apoptosis of MA-10 cells depended on ROS production.

The activation of p53 by therapeutic agents results in p53-mediated gene regulation (involving CDKIs, for example) to induce apoptosis and inhibit growth of cancer cells^43^. Our data showed that cordycepin upregulated levels of p53 and p21 and downregulated CDK2 in MA-10 cells. However, knockdown of p53 did not affect p21 or CDK2 levels in cordycepin-treated cells. Thus, alternative pathways activated by cordycepin may regulate p21 and CDK2. In fact, TGFβ could upregulate p21 through Smad4^44^. Here, cordycepin upregulated TGFβ2 and Smad4. It is therefore highly likely that p21 and CDK2 were regulated by p53 and/or TGFβ pathways to induce apoptosis in MA-10 cells.

**Figure 8:**
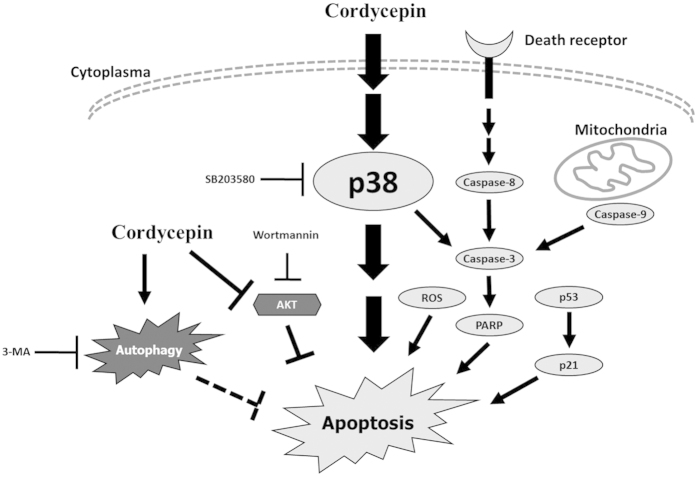
Schematic representation of cordycepin triggers apoptosis of MA-10 mouse Leydig tumor cells through p38, caspase, and PI3K/AKT/mTOR signaling pathways. Cordycepin treatment is able to induce the activation of p38. The activation of p38 is able to induce a cascade of events that include cleaved caspase-3 and PARP activation resulting in the induction of apoptosis. Cleaved caspase-3 also can be triggered by either death receptor-dependent or mitochondrion-dependent signaling pathways to promote the terminal phase of apoptosis. It should be also noted that treatment of cordycepin promotes MA-10 cell apoptosis through the activation of p53-dependent pathways and accumulation of ROS level. These signaling pathways associate and induce cell cycle arrest, leading to the final stages of apoptosis. Cordycepin also induced autophagy by suppressing PI3K/AKT/mTOR pathways. The molecular mechanism triggered by cordycepin in the MA-10 mouse Leydig tumor cells and this mechanism may be a model for future targets of cancer treatment. Potential major pathways are indicated by large arrowhead, and potential minor pathways are indicated by slight arrowhead.

Based on these results, we generated a model illustrating how cordycepin treatment is able to induce the apoptosis of MA-10 cells (Fig. 8). The activation of p38 is able to induce a cascade of events that include cleaved caspase-3 and PARP activation resulting in the induction of apoptosis. Cleaved caspase-3 also can be triggered by either death receptor-dependent or mitochondrion-dependent signaling pathways to promote the terminal phase of apoptosis. It should be also noted that treatment of cordycepin promotes MA-10 cell apoptosis through the activation of p53-dependent pathways and accumulation of ROS level. These signaling pathways associate and induce cell cycle arrest, leading to the final stages of apoptosis. Cordycepin also induced autophagy by suppressing PI3K/AKT/mTOR pathways. The molecular mechanism triggered by cordycepin in the MA-10 mouse Leydig tumor cells and this mechanism may be a model for future targets of cancer treatment.

Finally, we used allograft tumors to show *in vivo* that cordycepin reduced Leydig tumor growth, decreased levels of p-AKT, and increased levels of cleaved caspase-3. These results are similar to those achieved with cisplatin and bleomycin therapies on testicular germ-cell tumors^45^. We found no evidence of toxicity or side effects associated with cordycepin treatment, although side effects and resistance have been reported for cisplatin^46^. The inhibition of AKT and ERK in colorectal cancer cells prolongs survival in an allograft model of the disease^47^. Although these mechanisms were not explored in our model, cross-talk between different activators of AKT and ERK may have contributed to our findings. To our knowledge, this is the first study to assess the antitumor efficacy of cordycepin on testicular cancer *in vivo*.

In conclusion, we have demonstrated that cordycein induced MA-10 cell apoptosis via mediating p38 MAPKs signaling and reduced the growth of Leydig cancer cells *in vivo*. Specifically targeting PI3K/AKT/mTOR or autophagy pathways increased levels of cordycepin-induced apoptosis in MA-10 cells. These results indicate that cordycepin is a potential therapeutic agent for further development for the management of testicular cancer.

## Materials and Methods

### Chemicals

Cordycepin, taxol, cisplatin, etoposide, wortmannin, and methylthiazol tetrazolium (MTT) were purchased from Sigma-Aldrich (St. Louis, MO). Caspase inhibitors Z-VAD-FMK, Z-IETD-FMK, and Z-LEHD-FMK (which inactivate all caspases, caspase-8, or caspase-9, respectively) were purchased from R&D Systems (Minneapolis, MN). Antibodies against cleaved caspase-8 (Asp387), cleaved caspase-9 (Asp353), cleaved caspase-3 (Asp175), cleaved caspase-6 (Asp162), cleaved caspase-7 (Asp198), LC3 I/II, mTOR, phosphorylated mTOR (p-mTOR; Ser2448), AKT, p-AKT (Ser473), ERK, p-ERK (Thr202/Tyr204), JNK, p-JNK (Thr183/Tyr185), p38, p-p38 (Thr180/Tyr182), p53, and p-p53 (Ser15) were purchased from Cell Signaling (Beverly, MA). Antibodies against p21, p-p21 (Thr145), CDK2, TGFβ2, TGFβ3, and Smad4 were purchased from Santa Cruz Biotechnology (Santa Cruz, CA). Antibodies against β-actin were purchased from Sigma-Aldrich (St. Louis, MO), and antibodies against PARP were purchased from Oncogene (San Diego, CA).

### Cell culture

MA-10 cells were a gift from Dr. Mario Ascoli (University of Iowa, Iowa City, IA)^48^. Cells were maintained in Waymouth MB 752/1 medium (Sigma-Aldrich, St. Louis, MO) containing 10% fetal bovine serum (Invitrogen, Grand Island, NY) and incubated in a humidified atmosphere containing 95% air and 5% CO_2_ at 37 °C. The murine TM4 Sertoli-like cell line and NT2/D1 cells were obtained from the American Type Culture Collection (Manassas, VA)^49^. TM4 Sertoli cells were maintained in Dulbecco’s modified eagle medium, nutrient mixture F-12 (DMEM/F-12; Invitrogen, Grand Island, NY) containing 10% fetal bovine serum and incubated in a humidified atmosphere containing 95% air and 5% CO_2_ at 37 °C. NT2/D1 cells were maintained in Dulbecco’s modified eagle medium with high glucose (DMEM/HIGH GLUCOSE; Invitrogen, Grand Island, NY) containing 10% fetal bovine serum and incubated in a humidified atmosphere containing 95% air and 5% CO_2_ at 37 °C.

### Isolation of Leydig cells

Male B6 (C57BL/6NCrj) mice, 5–7 weeks old, were purchased from the National Cheng Kung University Animal Center (Tainan, Taiwan). All animals were housed in groups of four in 29 × 18 × 13–cm polyethylene cages. The animal room was maintained at 22–24 °C under a 12-h light/12-h dark cycle. Purina mouse chow (Ralston-Purina, St. Louis, MO) and water were provided *ad libitum*. The procedure for sacrificing animals was approved by the counselors of the National Cheng Kung University Animal Center. Testes were removed from sacrificed mice and decapsulated in M199 medium (pH 7.35) containing 4 mM NaHCO_3_, 25 mM HEPES, 0.06 g penicillin, 0.05 g streptomycin, and 0.2% bovine serum albumin (BSA). After decapsulation, testes were incubated in M199 medium containing 1% BSA and 100 U/mL collagenase for 15 min in a shaking water bath (120 cycles/min) at 37 °C. Cold M199 medium was then added to arrest the collagenase activity. Seminiferous tubules were separated from interstitial cells by gravity sedimentation. Cells were then collected by centrifugation (300 × *g*, 4 °C, 6 min) and resuspended in 2 mL M199 medium containing 0.1% BSA, to give an interstitial cell suspension containing 20–30% Leydig cells. A gradient of 10 mL isotonic Percoll solution and 15 mL M199 medium with 0.1% BSA and 25 mM HEPES was prepared by centrifugation (25 000 × *g*, 40 min). The interstitial cell preparation was layered onto the gradient and centrifuged (800 × *g*, 4 °C, 20 min). An 1-mL fraction of the gradient was collected from the top. Mouse Leydig cells were found primarily in fractions 23–25. 3 beta hydroxyl-delta-6-steroid dehydrogenase (3β-HSD) staining solution—which consisted of 1 mg/ml nitroblue tetrazolium, 3 mg/ml β-nicotinamide adenine dinucleotide, 2.88 mg/ml dihydroepiandrosterone in 10% acetone, and 1.6 mg/ml nicotinamide in 0.07 M phosphoric acid solution at pH 7.4—was used to identify the purity of Leydig cells. After isolation, cells were treated with 3β-HSD staining solution at 37 °C for 1 hr. The total number of cells and the percentage of 3β-HSD-positive cells were determined for the Leydig cell preparation^50^. The purity of the Leydig cells was reached 85–95%.

### MTT cell viability assay

MA-10 and TM4 cells were seeded into 96-well plates (Techno Plastic Products AG, Trasadingen, Switzerland) at 1 × 10^4^ cells in 100 μL medium per well. After reaching 70–80% confluence, cells were treated with cordycepin and/or kinase inhibitors. MTT was added to a final concentration of 0.5 mg/mL and the cells were incubated for 4 h at 37 °C. The medium was removed, 50 μL dimethyl sulfoxide were added per well, and crystals were dissolved by gently shaking in the dark for 20 min. Optical density at 570 nm (OD_570_) was determined using an ELISA microplate reader (VersaMax, Molecular Devices, Sunnyvale, CA).

### Lactate dehydrogenase (LDH) release assay

Primary mouse Leydig cells were seeded into 96-well plates at 5 × 10^4^ cells/well and incubated for 24 h at 37 °C in the presence or absence of cordycepin. LDH activity was measured in the culture medium using an LDH cytotoxicity assay kit (BioChain Institute, Heyward, CA). The red formazan product was measured at 490 nm using an ELISA microplate reader. Each treatment condition was assayed in triplicate wells. Mean values were expressed as a percentage of control (100% cytotoxicity).

### Annexin V staining and flow cytometry

MA-10 cells were treated with 10 μM, 100 μM, or 1 mM cordycepin for indicated amounts of time at 37 °C. Negative controls were incubated in plain medium, and positive control were incubated with 50 nM taxol for 24 h. Levels of apoptosis were determined by incubating cells with fluorescently labeled annexin V (Annexin V-FITC apoptosis detection kit, Strong Biotech, Taipei, Taiwan). After incubation with annexin V, floating and adherent cells were trypsinized, pooled, and centrifuged (1 000 × *g*, 10 min). Pelleted cells were washed in cold phosphate-buffered saline (PBS), centrifuged again (1 000 × *g*, 10 min), and resuspended in 100 μL of 1× annexin-binding buffer (10 mM HEPES, 140 mM NaCl, 2.5 mM CaCl_2_, pH 7.4) to yield a cell density of 1 × 10^6^ cells/mL. Next, 2 μL annexin V conjugate and 2 μL of 100 μg/mL propidium iodide (PI) working reagent were added to each 100 μL cell suspension and cells were incubated at room temperature for 15 min. Finally, 500 μL of 1× annexin-binding buffer was added, and cells were gently mixed. Labeled cells were analyzed by flow cytometry with fluorescence emissions measured at 515 nm (FACScan, Becton-Dickinson, Mountain View, CA). The percentage of cells was calculated using Cell-Quest software (Becton-Dickinson, Mountain View, CA).

### Western blotting

MA-10 cells were cultured in 6-cm dishes at 6 × 10^5^ cells in 2 mL medium. After treatment, cells were rinsed with cold PBS and harvested using 100 μL lysis buffer (20 mM Tris-base, 150 mM NaCl, 1 mM ethylenediaminetetraacetic acid, 1 mM ethylene glycol tetraacetic acid, 1% Triton X-100, 2.5 mM sodium pyrophosphate, 1 mM β-glycerophosphate, 1 mM Na_3_VO_4_). The lysate was centrifuged (12 000 × *g*, 12 min, 4 °C) and the supernatant stored at −20 °C for later use. Protein concentration was determined using a Micro BCA protein assay kit (Thermo Fisher Scientific, Waltham, MA). Total proteins were separated by sodium dodecyl sulfate (SDS) polyacrylamide gel electrophoresis, using a 12.5% gel and a running buffer of 24 mM Tris-HCl, 0.19 M glycine, 0.5% SDS, pH 8.3. Proteins were transferred to polyvinylidene difluoride membranes in transfer buffer (20 mM Tris-HCl, 150 mM glycine, 10% methanol, 0.05% SDS). The membranes were incubated in blocking buffer (Tris-buffered saline containing 5% nonfat dry milk and 0.1% Tween-20) at room temperature for 1 h. After washing, the membranes were incubated with primary antibodies (1:4 000) overnight at 4 °C. The membrane was washed three times (10 min each) with Tris-buffered saline containing 0.1% Tween-20, and then incubated with horseradish peroxidase–conjugated antibodies against mouse or rabbit. The immunocomplex was visualized using an enhanced chemiluminescence detection kit (EMD Millipore Corporation, Billerica, MA), and protein levels were quantified using a UVP EC3 imaging system (Upland, CA). β-actin served as a loading control.

### ROS measurements

At the desired time points, control and treated cells were trypsinized and resuspended in phenol red–free Waymouth MB 752/1 medium with 2 μM 5-(and-6)-chloromethyl-20,70-dichlorodihydrofluorescein diacetate acetyl ester (DCF-DA; Sigma-Aldrich, St. Louis, MO). Cells were incubated with DCF-DA at 37 °C for 30 min, with 1 μM Hoechst stain (Invitrogen, Grand Island, NY) added for the final 5 min. Stained cells were washed, resuspended in ice-cold PBS, and immediately analyzed using a BD FACSCanto II flow cytometer (515–545 nm) with BD FACSdiva software (Becton-Dickinson, Mountain View, CA) or by fluorescence microscopy (Nikon, ECLIPSE TS100, Tokyo, Japan).

### Small interfering RNA (siRNA) design and transient transfection

Block-iT RNAi design software (Invitrogen, Grand Island, NY) was used to design siRNAs targeting p53 (CAC CUC ACU GCA UGG ACG AUC UGU U or GAG UAU CUG GAA GAC AGG CAG ACU U or GGG ACA GCU UUG AGG UUC GUG UUU G) and caspase-3 (UUA UUA UGC AUA UGC CCA UUU CAG G or UAG AAU CAC ACA CAC AAA GCU GCU C or ACU ACU GCC GGA GUC UGA CUG GAA A). For transfections, 2.0 × 10^5^ MA-10 mouse Leydig tumor cells were seeded into 6-well plates and grown to 60–70% confluency without antibiotics. Cells were transfected with siRNAs (10 nM) using 7.5 μL lipofectamine RNAiMAX (Invitrogen, Grand Island, NY) in a total volume of 2.5 mL. Cells were transfected with siRNAs for 6 h, then the transfection medium was replaced with fresh Waymouth medium containing 10% FBS, and cells were incubated for an additional 24 h before protein samples were collected for western blotting. Each siRNA experiment was performed in triplicate. A negative control involving a siRNA that was not homologous to any known genes (Thermo Fisher Scientific, Waltham, MA) was used to control against nonspecific effects of oligonucleotides. The transfection efficiency associated with each siRNA was confirmed using SiGLO Red transfection indicators (Thermo Fisher Scientific, Waltham, MA). Uptake of the fluorescent oligos, which correlates with uptake of Stealth™ RNAi, was used to measure transfection efficiency.

### Animal treatment

Male CB17/Icr-Prkdc^scid^/CrlNarl and severe combined immunodeficiency (SCID) mice (12 weeks old) were purchased from National Cheng Kung University Animal Center (Tainan, Taiwan). To induce tumor growth, mice were injected subcutaneously with 1 × 10^7^ MA-10 mouse Leydig tumor cells suspended in 0.1 mL PBS. Tumors were allowed to grow for 1 week and mice were randomly assigned to vehicle control or cordycepin treatment (6 mice per group). Injections (0.1% cordycepin in PBS; 20 mg/kg) were performed once daily. Tumors were measured once daily and tumor volumes were calculated using the formula: length × width × depth × 3.14/6. Data were expressed as average tumor volume ± SEM. After animals were sacrificed, tumors were harvested to evaluate protein localization by immunohistochemistry. We have followed the ARRIVE guidelines regarding animal studies^51^.

### Ethics statement

The animal study is approved by the institutional animal care and use committee (IACUC) of National Chen Kung University with approval No. 100111. The methods were carried out in accordance to the approved guidelines.

### Immunohistochemistry

Mice were perfused with cold PBS (pH 7.4) followed by 4% paraformaldehyde in PBS. Allografted tumors were removed and cryoprotected in PBS containing 30% sucrose. The tumor blocks were serially sectioned on a cryostat (Leica Microsystem, Wetzlar, Germany) with section thickness ranging from 20 to 30 μm. Tissue sections were prepared and then incubated in blocking buffer (containing goat serum) followed by primary antibodies (cleaved caspase-3 or p-AKT) for 2 h. Sections were washed and then incubated with rhodamine-conjugated secondary antibodies (Jackson ImmunoResearch Laboratories, West Grove, PA). Nuclear counterstaining was performed with hematoxylin. Immunolabeled sections were mounted and observed by fluorescence microscopy. For DAB staining and immunohistological analysis, cryoprotected tumor sections were incubated in blocking buffer (containing donkey serum) and then incubated with primary antibodies (cleaved caspase-3 or p-AKT). Sections were then washed and incubated with biotinylated donkey secondary antibodies followed by avidin-biotin complexes (ABC). Peroxidase labeling was performed using a DAB substrate kit (Vector Laboratories, Burlingame, CA). Sections were mounted with DPX mounting solution (BDH, Atlanta, GA) and examined using a light microscope (Zeiss, AX10, Thornwood, NY).

### Statistical analysis

Within each figure, data represent mean ± SEM of three or four independent experiments. Statistically significant differences between groups were determined using the Student’s t-test or one-way analysis of variance and the least significant difference. Statistical significance was set at *p* < 0.05.

## Additional Information

**How to cite this article**: Pan, B.-S. *et al.* Cordycepin induced MA-10 mouse Leydig tumor cell apoptosis by regulating p38 MAPKs and PI3K/AKT signaling pathways. *Sci. Rep.* **5**, 13372; doi: 10.1038/srep13372 (2015).

## Supplementary Material

Supplementary Information
