# Supplementary material for: Cordycepin induced MA-10 mouse Leydig tumor cell apoptosis by regulating p38 MAPKs and PI3K/AKT signaling pathways
Source: Sci Rep. 2015 Aug 25;5:13372. doi: 10.1038/srep13372 (PMC4548195; doi:10.1038/srep13372)
Supplement: Supplementary Information [file srep13372-s1.doc]

**Supplementary materials**

**Cordycepin-induced MA-10 Mouse Leydig Tumor Cells apoptosis by regulating p38 MAPKs and PI3K/AKT signaling pathways**

Bo-Syong Pan^1,2^, Yang-Kao Wang^1,2^, Meng-Shao Lai^1,2^, Yi-Fen Mu^2^, Bu-Miin Huang^1,2^,*

^1^Institute of Basic Medical Sciences and ^2^Department of Cell biology and Anatomy, College of Medicine, National Chen Kung University, Tainan, Taiwan, Republic of China

*Corresponding author:

Bu-Miin Huang, Ph.D., Department of Cell Biology and Anatomy, College of Medicine, National Cheng Kung University, #1 University Road, Tainan, 70101, Taiwan, Republic of China, Tel: 886-6-2353535 ext. 5337, Fax: 886-6-209-3007, E-mail: [bumiin@mail.ncku.edu.tw](mailto:bumiin@mail.ncku.edu.tw)

**(A).**

**(B).**

**
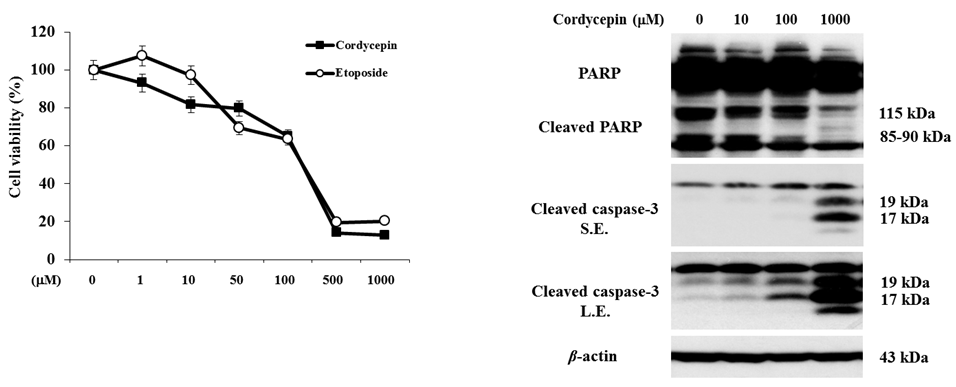
**

**(C).**

**(D).**


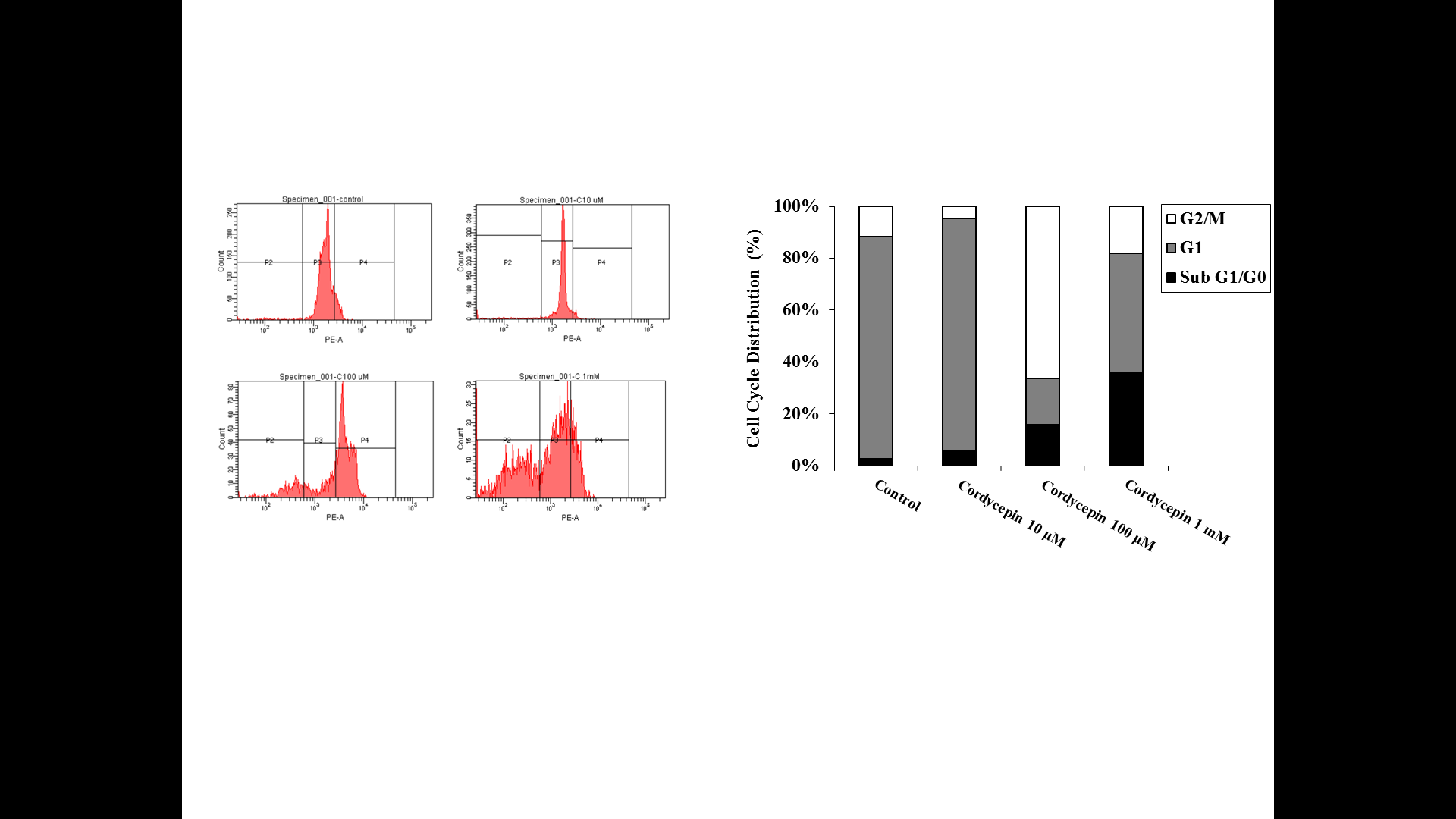


**Figure S1. Cordycepin induced apoptosis of testicular germ cell tumors.** (A) NT2/D1 cells were treated with or without cordycepin and etoposide at the indicated dosages for 24 h. (B) NT2/D1 cells were treated with or without cordycepin for 24 h. Levels of cleaved caspase-3 (17/19 kDa) and cleaved PARP (85-90 kDa) were detected by western blotting. (C) NT2/D1 cells were treated with or without cordycepin at the indicated dosages at 24 h, and stained with propidium iodide (PI). Flow cytometry was used to determine the fractions of sub G1/G0, G1, and G2/M phases. (D) Plot of cell cycle distribution from flow cytometric analysis from (C).

**(A).**

**(B).**


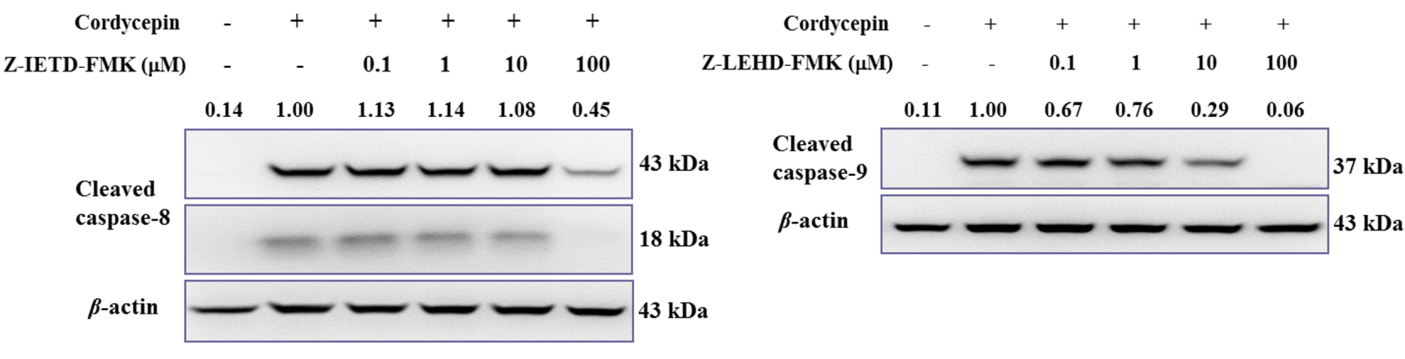


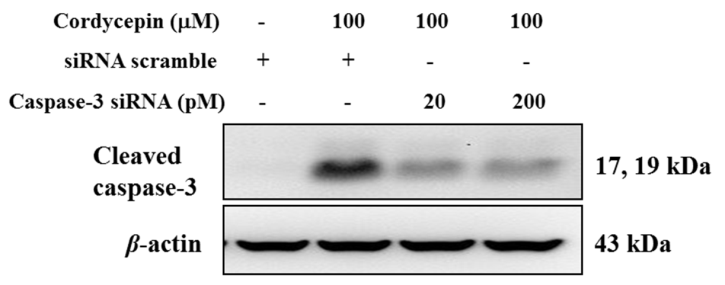

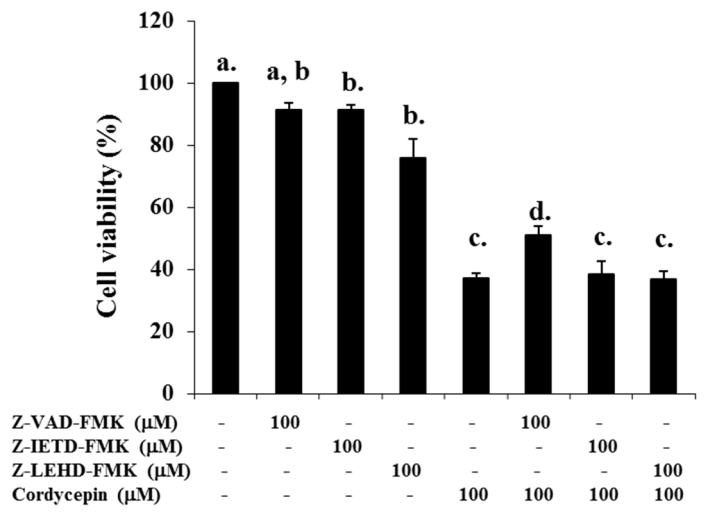


**(C).**

**(D).**

**(E).**

**(F).**


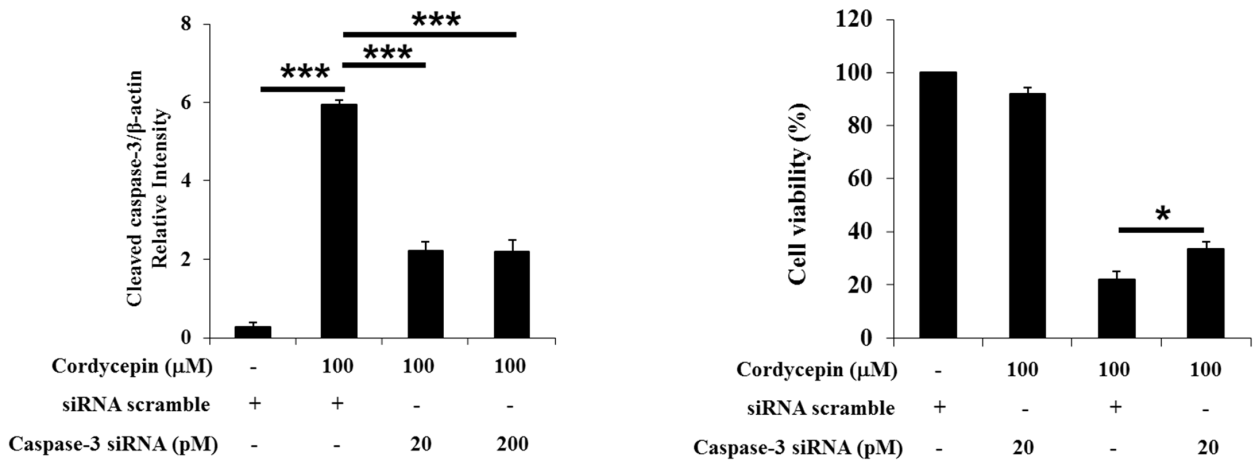


**Figure S2.** Effect of caspase-3 signaling pathway in cordycepin-induced cell death in MA-10 cells (related to Figure 2). (A-B) MA-10 cells were pretreated with the caspase-8 inhibitor Z-IETD-FMK (A) or the caspase-9 inhibitor Z-LEHD-FMK (B) for 1 h, then 100 μM cordycepin was added and cells were treated for 12 h. Cleaved caspases were detected by western blotting. Band intensities relative to β-actin are shown above each blot. (C) Cell viability was analyzed using the MTT assay after cells were treated with the indicated caspase inhibitors and 100 μM cordycepin for 24 h. (D) MA-10 cells were treated with 100 μM cordycepin and/or caspase-3 siRNA (20 or 200 pM) for 24 h. Cleaved caspase-3 were detected by western blotting. (E) Integrated optical densities of protein bands in (D). (F) Cell viability was analyzed using the MTT assay after cells were treated with 100 μM cordycepin and/or caspase-3 siRNA (20 pM) for 24 h. Data represent mean ± SEM of three independent experiments. Different letters (a−d) above the bar indicate the significant differences among each treatment (p < 0.05). *, *p* < 0.05; ***, *p* < 0.001.

**
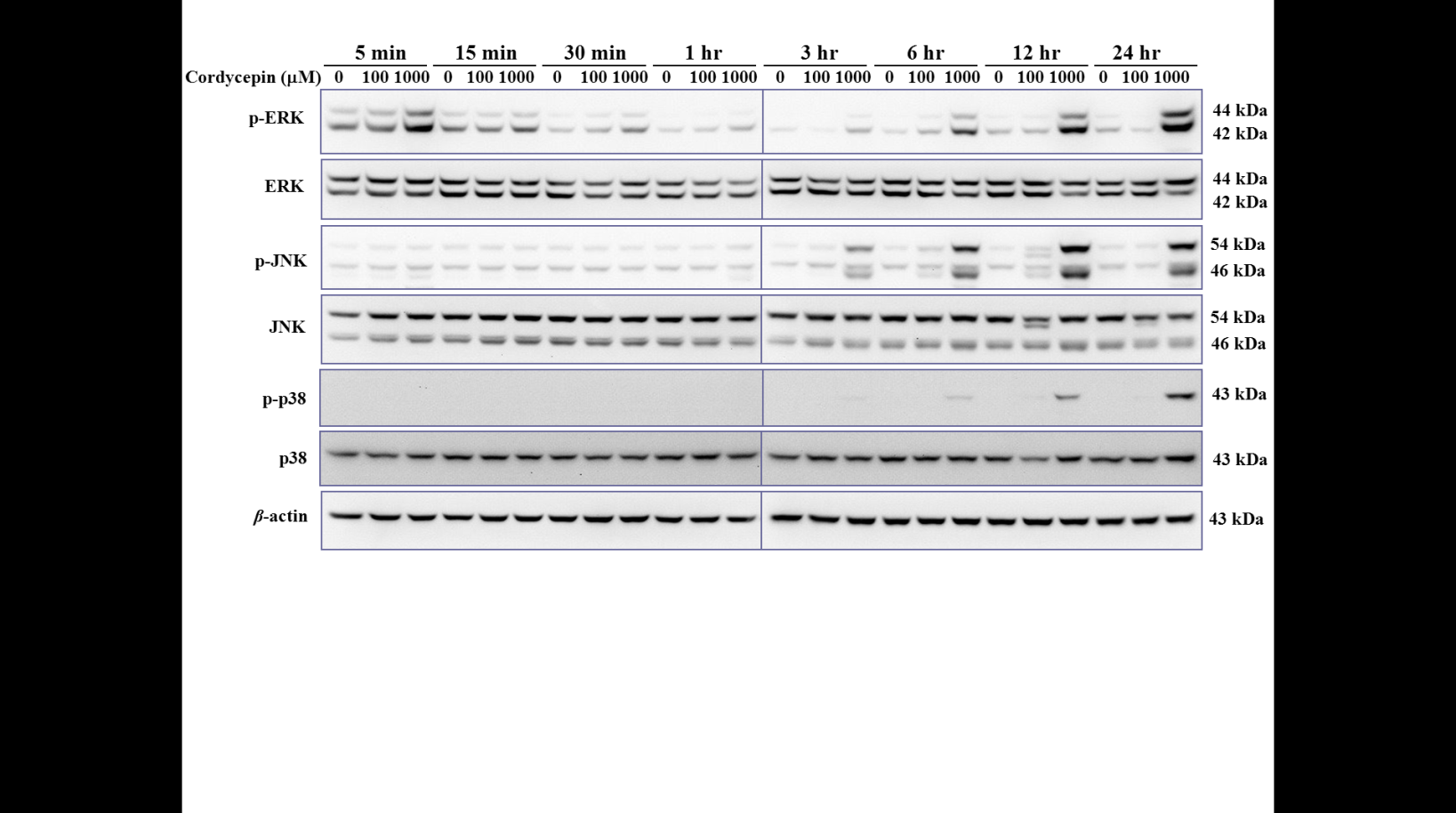
**

**(A).**

**(B).**


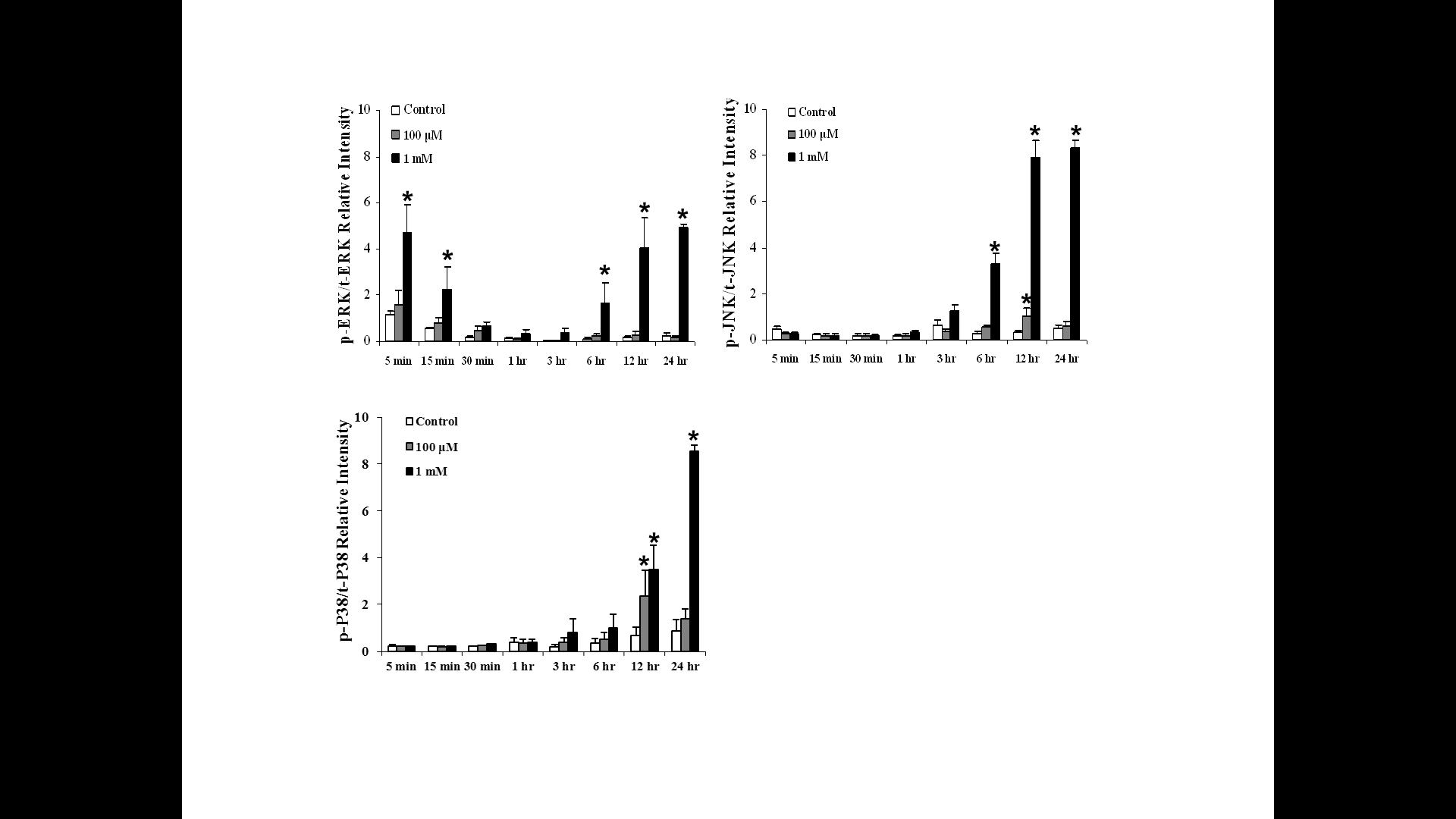

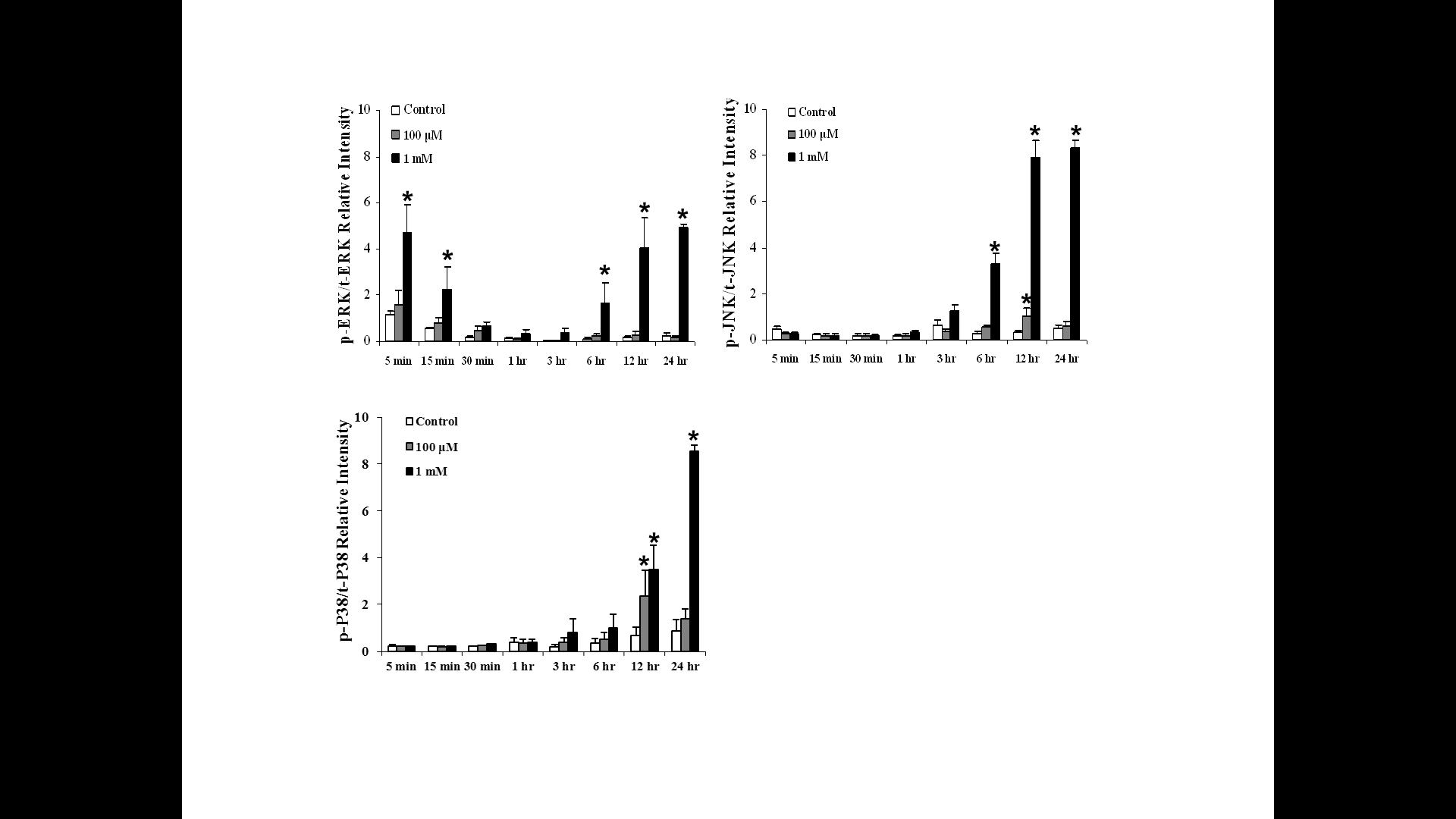


**(C).**

**(D).**


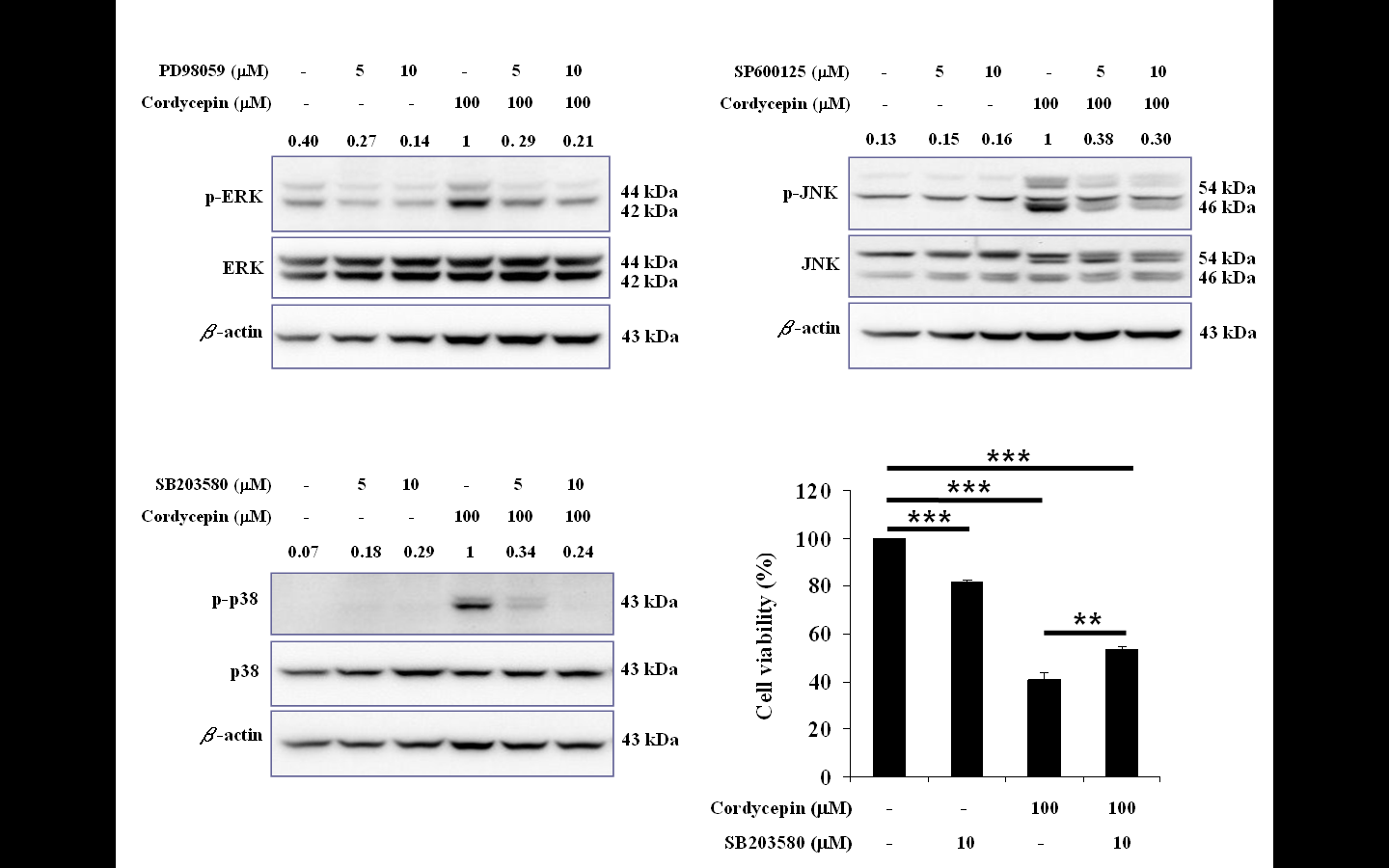


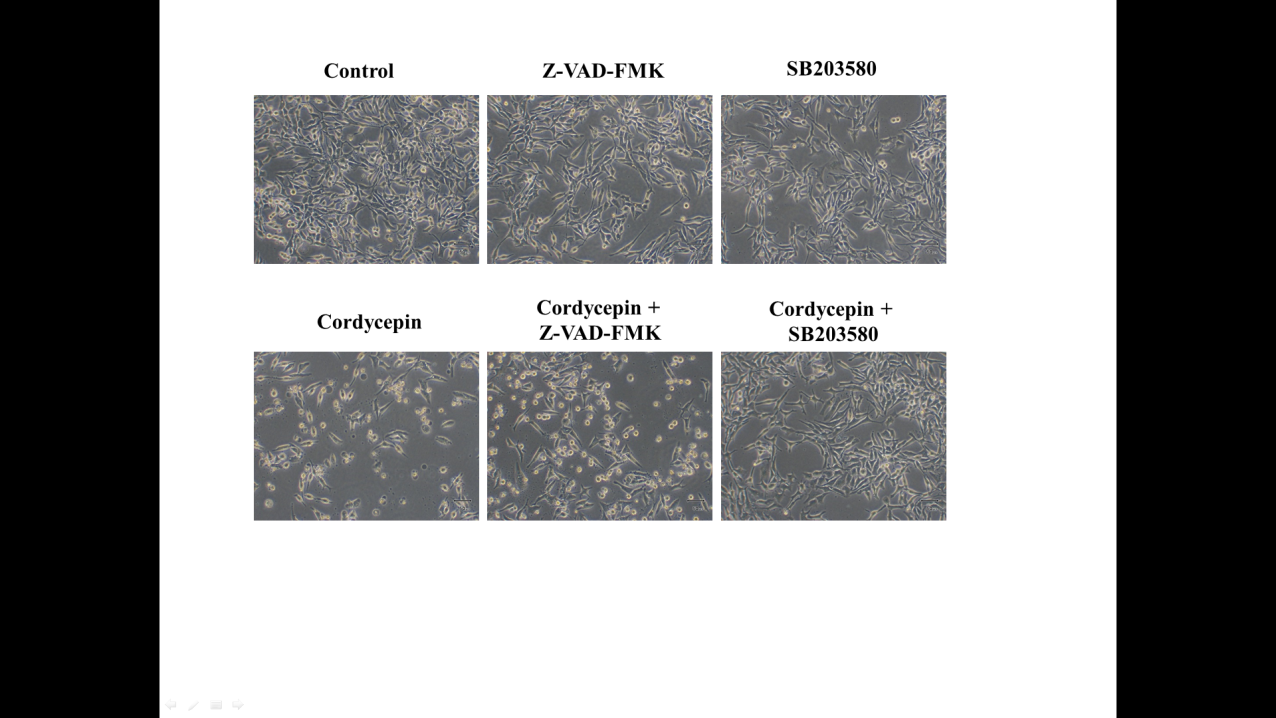

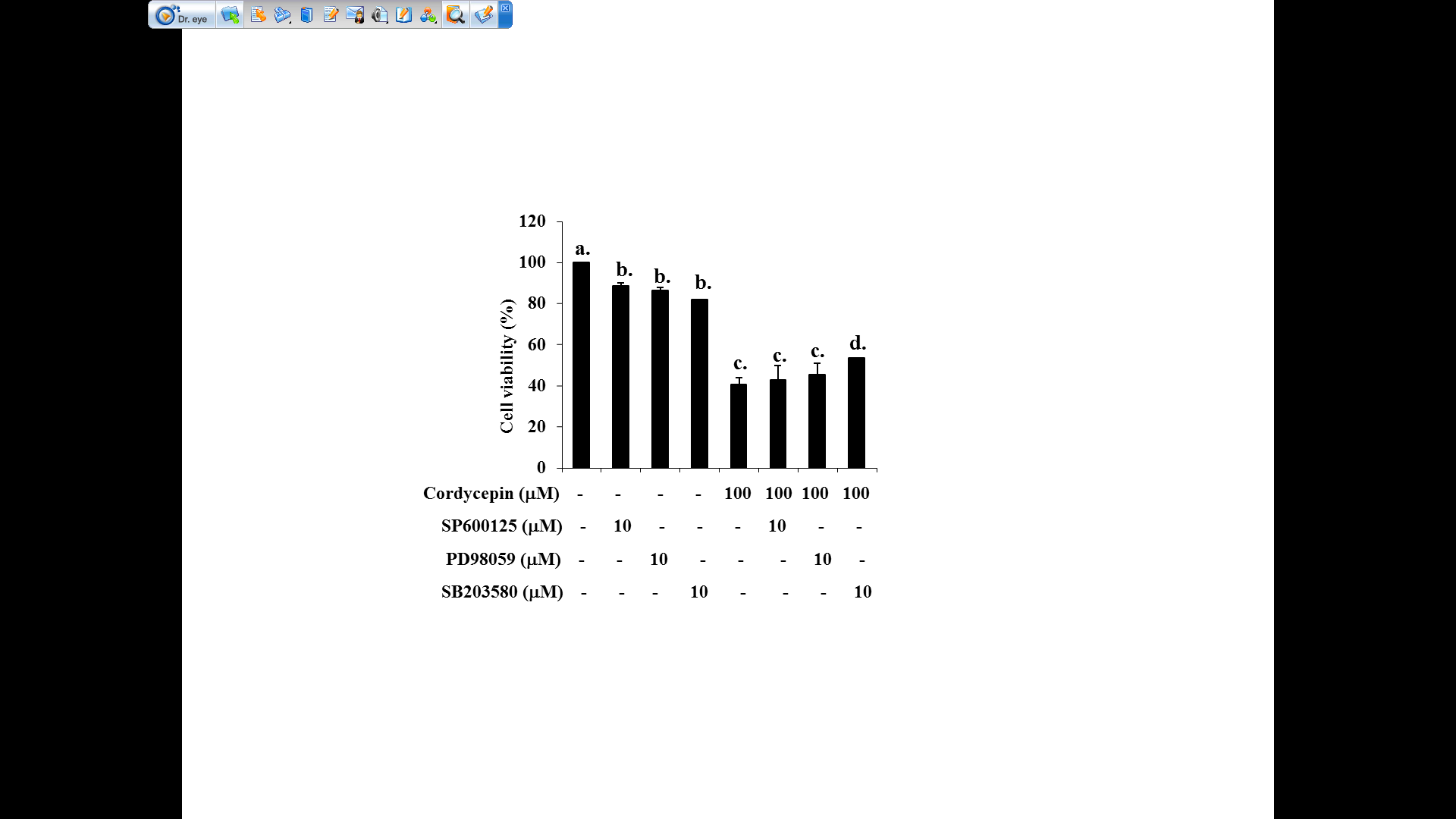


**(E).**

**(F).**

**Figure S3.** Cordycepin induced MAPK signaling activation in MA-10 cells (related to Figure 3). (A) MA-10 cells were treated with or without cordycepin for the indicated times. Total and phosphorylated ERK (44 and 42 kDa), JNK (54 and 46 kDa), and p38 (43 kDa) were detected by western blotting. (B) Integrated optical densities of proteins bands in (A). Data were normalized to respective total proteins. (C–D) MA-10 cells were pretreated with ERK inhibitor PD98059 (C) and JNK inhibitor SP600125 (D) for 1 h, then 100 μM cordycepin was added and cells were treated for 12 h. Total and phosphorylated proteins were detected by western blotting. Band intensities relative to β-actin are shown above each blot. (E) Cell viability was analyzed using the MTT assay after cells were treated with indicated MAPKs inhibitors without or with 100 μM cordycepin for 24 h. (F) Phase contrast images of cells that were treated with general caspase inhibitor Z-VAD-FMK and p38 inhibitor SB203580 with or without cordycepin for 24 h. Different letters (a−d) above the bar indicate the significant differences among each treatment (p < 0.05). *, *p* < 0.05
